# A Transformer-Based Neural Network for Gait Prediction in Lower Limb Exoskeleton Robots Using Plantar Force

**DOI:** 10.3390/s23146547

**Published:** 2023-07-20

**Authors:** Jiale Ren, Aihui Wang, Hengyi Li, Xuebin Yue, Lin Meng

**Affiliations:** 1 School of Electronic and Information, Zhongyuan University of Technology, Zhengzhou 451191, China; 2021106221@zut.edu.cn; 2 Research Organization of Science and Technology, Ritsumeikan University, Kusatsu 525-8577, Japan; lihengyi@fc.ritsumei.ac.jp (H.L.); yue-xb@fc.ritsumei.ac.jp (X.Y.); 3 College of Science and Engineering, Ritsumeikan University, Kusatsu 525-8577, Japan; menglin@fc.ritsumei.ac.jp

**Keywords:** gait prediction, transformer, convolutional neural network, plantar pressure

## Abstract

Lower limb exoskeleton robots have shown significant research value due to their capabilities of providing assistance to wearers and improving physical motion functions. As a type of robotic technology, wearable robots are directly in contact with the wearer’s limbs during operation, necessitating a high level of human–robot collaboration to ensure safety and efficacy. Furthermore, gait prediction for the wearer, which helps to compensate for sensor delays and provide references for controller design, is crucial for improving the the human–robot collaboration capability. For gait prediction, the plantar force intrinsically reflects crucial gait patterns regardless of individual differences. To be exact, the plantar force encompasses a doubled three-axis force, which varies over time concerning the two feet, which also reflects the gait patterns indistinctly. In this paper, we developed a transformer-based neural network (TFSformer) comprising convolution and variational mode decomposition (VMD) to predict bilateral hip and knee joint angles utilizing the plantar pressure. Given the distinct information contained in the temporal and the force-space dimensions of plantar pressure, the encoder uses 1D convolution to obtain the integrated features in the two dimensions. As for the decoder, it utilizes a multi-channel attention mechanism to simultaneously focus on both dimensions and a deep multi-channel attention structure to reduce the computational and memory consumption. Furthermore, VMD is applied to networks to better distinguish the trends and changes in data. The model is trained and tested on a self-constructed dataset that consists of data from 35 volunteers. The experimental results show that FTSformer reduces the mean absolute error (MAE) up to 10.83%, 15.04% and 8.05% and the mean squared error (MSE) by 20.40%, 29.90% and 12.60% compared to the CNN model, the transformer model and the CNN transformer model, respectively.

## 1. Introduction

A lower limb exoskeleton robot is a kind of wearable robot. In rehabilitation therapy, the robot is designed for patients with lower limb motor dysfunction, providing efficient and safe assistance in their rehabilitation training [1,2,3]. In the manufacturing industry, it is capable of enhancing the motor ability of wearers by reducing the energy consumption, enabling users to perform high-intensity work with greater ease [4,5]. Due to its pivotal role in addressing population aging and intensive industrial transformations, the field of lower limb exoskeletons has a substantial research value. While significant progress has been made in exoskeleton technology [6,7,8], the optimization of human–robot collaboration remains a persistent challenge due to the intricate interactions between the wearers and the exoskeletons [9]. In particular, accurately predicting wearers’ motion intentions, thus optimizing the controlling strategy, has consistently been a critical issue and remains a prominent area of exploration in the field of exoskeletons.

Gait data, encompassing physical quantities such as plantar pressure, angles and torques of the hip joint, knee joint and ankle joint, as well as biological signals like electromyography (EMG) and electroencephalography (EEG) signals, serve as the fundamental basis for human walking pattern analyses and motion intention predictions. EMG and EEG are capable of reflecting the motion intention with a low delay. However, the measurement of bioelectrical signals requires electrodes to be placed close to the skin surface, which inevitably causes discomfort to the subject. Moreover, these signals exhibit great randomness and a relatively low signal-to-noise ratio. Joint angles and torques are easy to measure using absolute encoders and joint drivers installed in robots. However, physical signals obtained by sensors often contain a certain delay and thus cause lagging control commands.

The plantar pressure is one of the most important physical signals [10]. During walking, the hip and knee joints first flex to lift the thighs forward, bring the calves closer to the hips and then extend the hip joint to straighten the legs. Dorsiflexion of the ankle joint lifts the toes off the ground and helps the foot complete the swing, while plantarflexion positions the toes on the ground, lifting the heel. Through the synergism of lower limb joints, the body’s weight, kinetic energy and muscle force are transmitted to the plantar, generating plantar pressure and pushing the body forward. Meanwhile, the ground exerts counterforce on the foot, helping maintain the balance and stability of the body. Given the close connection between plantar pressure and joint movement, collecting plantar pressure signals to predict the human gait trajectory is an effective method that not only compensates for the measurement lag but provides references for robot control.

When comparing statistical methods [11,12,13], deep learning methods demonstrate a better feature extraction performance, especially for non-stationary series and multivariate time series forecasting. The mechanism of parameter sharing grants recurrent neural networks (RNNs) an edge in processing sequential data [14,15,16]. Convolutional neural networks (CNNs) are powerful in feature extraction, especially for data with a grid structure [17,18,19,20,21]. Transformers completely rely on an attention mechanism to characterize the global dependency relationship between the input and output of the model, featuring superiority in capturing the long-term dependence of sequence data [22]. Due to the high memory usage and time complexity, the basic model of the transformer needs to be improved in practical applications.

Time series can be visualized in the time domain, but mere time domain analyses cannot accurately capture the intrinsic patterns of change in the data due to the mix of trends, periodicity and nonlinearity. Frequency domain analysis methods, such as empirical mode decomposition (EMD) [23] and variational mode decomposition (VMD) [24], which decompose data according to their frequency characteristics, are commonly used in data processing to describe data features more effectively. Compared to EMD, VMD is much more robust to sampling and noise, thus it is often used in prediction tasks as part of data analysis and processing.

In view of the above points, we develop a novel transformer-based neural network named TFSformer for the precise prediction of hip and knee joint angles utilizing plantar pressure data. There are several primary structures designed in TFSformer. (1) We construct a 1D convolution-based encoder to extract essential information in both temporal and force-space dimensions. (2) We design a multi-channel attention mechanism, inspired by the multi-head attention mechanism employed in transformers. We extend the data by one dimension and define this as the channel dimension. Then, the size of the channel dimension is incrementally increased by a 2D convolution operation, and each sublayer within the channel dimension serves as a single attention head. (3) The deep multi-channel attention structure is further constructed by adding a pooling layer between two multi-channel attention layers, which is capable of reducing the memory usage and calculation consumption while retaining important information. In addition, the original data are decomposed by VMD before being input into the decoder part of the model. Our main contributions are summarized as the following:

- We propose a gait prediction method in terms of plantar pressure data and deep learning. The predicted joint angles are capable of compensating for the sensor delay and serving as a reference for the controller.
- We introduce the transformer-based model, TFSformer, which effectively integrates features from both temporal and force-space dimensions. The proposed model features a high performance in gait prediction tasks, achieving the minimum mean absolute errors of 0.0683, 0.0685 and 0.0677 in three tasks set in our experiment, respectively.
- We construct a dataset containing data from 35 volunteers, providing a data foundation for network training and evaluation.

The rest of this paper is organized as follows: Section 2 presents a comparative review of the relevant research on gait patterns for lower limb exoskeleton robots and transformer-based networks for time series issues. Section 3 introduces the gait pattern dataset established for the research and provides detailed principles of the theory basis. Section 4 presents the architecture of the proposed model. Section 5 gives the details of the experiment setup and experiment results. Finally, Section 6 summarizes the research work and outlines future plans.

## 2. Related Work

### 2.1. Gait Analysis for Lower Limb Exoskeleton Robots

Gait analyses play a pivotal role in the field of lower limb exoskeletons with two primary objectives: providing human factor references for controller design and adjusting the reference trajectory of the robot based on the wearer’s motion intention. Numerous studies have been dedicated to analyzing gait in this context to further advance the development of lower limb exoskeletons.

To address the time delay problem of sensor measurements, human gait information and patterns are learned based on a Gaussian process model to predict joint angles [25]. The predicted angles and predicted uncertainty indicators were set as the reference parameters of the variable conductance controller by Chen et al. Based on the joint angle data, walking speed and body parameters, Wu et al. established mapping relationships between the body parameters and the gait features to generate individualized gait patterns [26]. Utilizing the angle of hip and knee joints and plantar pressure data, Wu et al. used a graph convolutional network model to classify the gait phase for a lower limb exoskeleton robot [27]. For children with cerebral palsy, Kolaghassi et al. implemented four deep learning models (LSTM, FCN, CNN and a transformer) to predict joint angles and proposed an approach for adaptive trajectory generation based on a dataset consisting of flexion–extension angles of the hip, knee and ankle in the sagittal plane [28]. Huang et al. attached an intelligent inertial measurement unit (IMU) to the surface of a shoe to sample the acceleration data of foot movements and proposed an online detection algorithm to identify the gait phase [29]. For the speed adaptation control of exoskeletons, Zhang et al. proposed a method for walking speed learning and generalization, combining the advantages of RNNs and a sequence-to-sequence structure [30]. In simulation and offline experiments, they used sinusoidal signals and walking data from three subjects to evaluate the performance of the model.

As indicated above, deep learning has become a mainstream method in gait analyses for its superiority in handling non-linear problems. Furthermore, compared to RNNs and CNNs, transformer-based models are relatively less used in gait analyses.

### 2.2. Transformer-Based Network for Time Series Issue

Sensor data are a type of time series data. Processing methods in time series issues are able to provide a reference for handling sensor data.

Wang et al. applied a transformer model to predict stock markets [31]. The results showed that the transformer exhibits a higher prediction accuracy than traditional deep learning models like CNNs and RNNs. The network proposed by Chen et al. for multivariate time series anomaly detection uses a transformer-based architecture to model the temporal dependency [32]. Kim et al. proposed a prediction-based unsupervised time series anomaly detection using a transformer [33]. The encoder, modeled by transformer layers, accumulates the hierarchical information from the sequence model, while they utilized a one-dimensional convolution operation to replace the original decoder architecture to extract common information. The temporal fusion transformer (TFT) is a multi-scale prediction model incorporating a static covariate encoder, a gated selection method and a self-attention mechanism to capture short-term and long-term time correlations [34]. In the TFT, LSTM is used for local processing and multi-head attention is used to integrate information. Based on the Probsparse self-attention mechanism and the self-attention distilling operation, an informer reduces the time complexity and memory usage compared to a transformer [35]. An autoformer decomposes the initial data into seasonal and trend cyclical parts using an auto-correlation mechanism to extract valid information [36]. The Fourier-enhanced block and wavelet-enhanced block in the FEDformer were used to capture important structures in time series through frequency domain mapping [37].

The studies above demonstrate that the transformer structure is suitable for time series problems and features a strong flexibility, which is able to be combined with other networks. Most transformer variants retain the core characteristics, i.e., a sequence-to-sequence structure and an attention mechanism. Similarly, in this article, we construct a transformer-based network to predict the angles of hip and knee joints.

## 3. Preliminary

This section introduces the work undertaken in relation to the construction of the dataset used in this paper firstly, then elucidates the methodologies and theoretical frameworks that underpin our proposal, providing a thorough understanding of the conceptual and methodological foundations upon which the proposed model is built.

### 3.1. Data Acquisition and Preprocessing

#### 3.1.1. Data Acquisition

**Figure 1:**
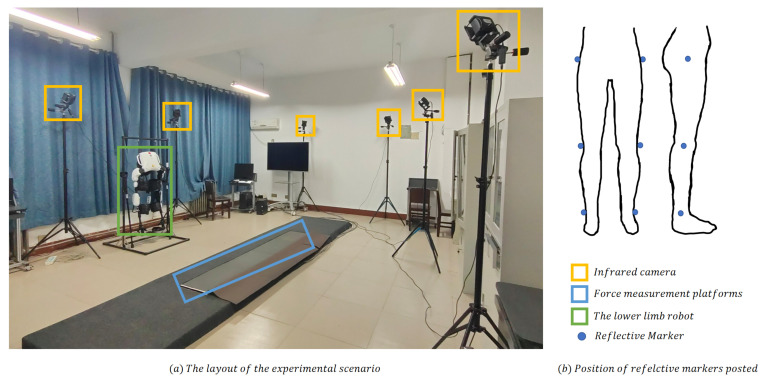
The layout of the experimental scenario and position of reflective markers.

The dataset for the research consists of joint angle data and plantar pressure data associated with the process of human walking. To establish a precise and comprehensive dataset, we designed an experimental setup utilizing state-of-the-art equipment, including the high-precision Nokov 3D infrared passive optical capture system in combination with four Bertec force measurement platforms. This meticulously constructed experimental scenario allowed us to accurately capture and analyze the desired data points, ensuring a robust and reliable foundation for the research. Figure 1a showcases the experimental scenario: eight infrared cameras are placed close to the wall, forming an oval shape to surround the measurement area and four force measurement platforms are meticulously positioned in a sequential arrangement at the center of the measurement area.

A total of 35 healthy volunteers were recruited to participate in the gait acquisition experiments. During the data collection experiments, the subjects were instructed to maintain their hands by their sides to avoid obstructing the markers, and they walked at their self-perceived most comfortable speed and stride length, ensuring the natural pattern of the collected gait data. The reflective markers, which were captured by infrared cameras, were affixed to the hip joints, knee joints and ankle joints of subjects. Figure 1b depicts the marker placements which were meticulously arranged to accurately capture the kinematic data for the gait analysis.

#### 3.1.2. Data Preprocessing

The raw data obtained from the experimental equipment inevitably exhibit certain limitations or imperfections. Thus, the raw data were further preprocessed with the following principles to ensure the integrity and accuracy of the dataset:

- For data with small portions missing, typically not exceeding five samples, cubic spline interpolation was applied to fill the data and ensure data continuity.
- For the data with severely missing values or excessive noise, all data were deleted to ensure the availability of reliable data.
- To further reduce the noise and enhance the quality of the data, a filtering operation with a cut-off frequency of 60 Hz was applied.

These procedures were rigorously implemented to ensure that the dataset obtained for this research was of the utmost quality, ensuring the integrity and accuracy of the research findings.

**Figure 2:**
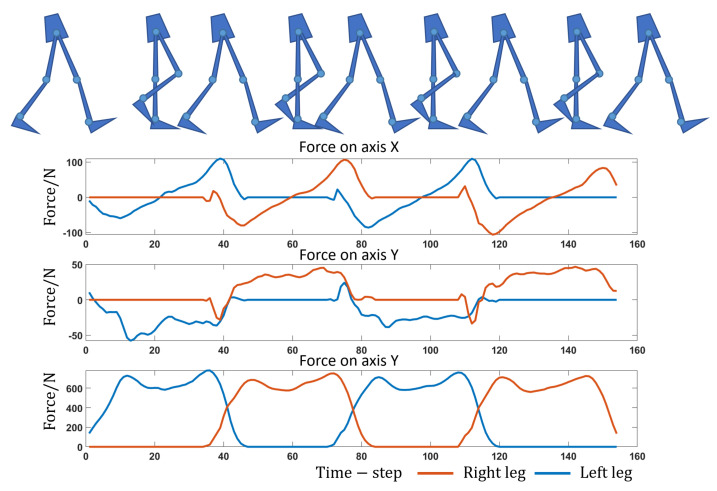
Plantar pressure.

Figure 2 displays the bilateral plantar pressure curves and corresponding walking posture. During walking, the feet alternately touch the ground and the body’s gravity shifts from one lower limb to the other, the plantar force changing with the gravity change.

**Figure 3:**
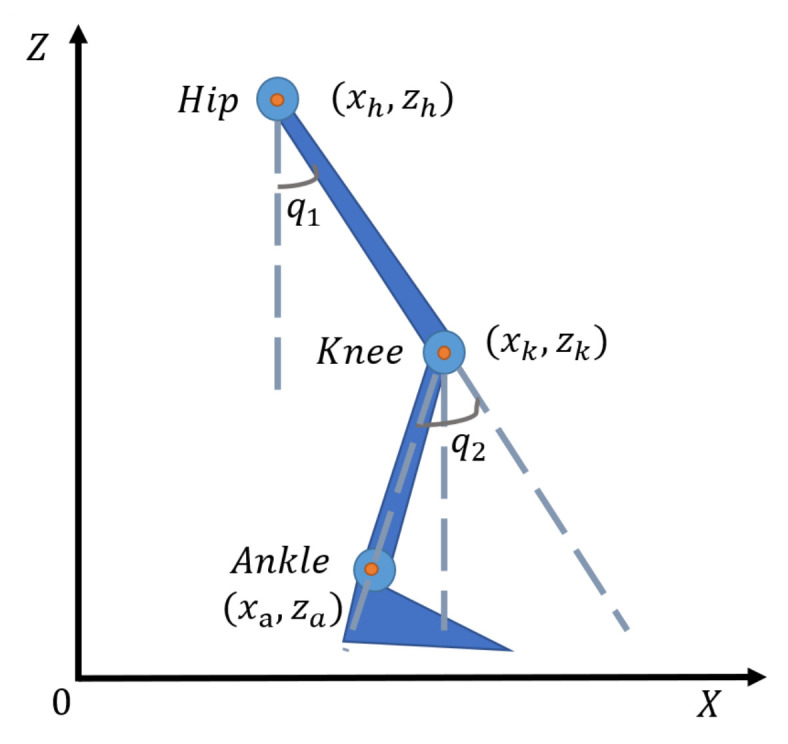
Two-link model.

The data collected by the motion capture device are joint coordinate data, while joint angle data are more applicable in the design of controllers. Therefore, the joint angle data were further calculated using the inverse dynamics kinematic principle. As shown in Figure 3, the lower limb can be simplified to a two-link model. The hip angle and the knee angle were set to $q_{1}$, and $q_{2}$, respectively. The coordinate data of hip, knee and ankle joints were set to $\left(x_{h},z_{h}\right)$, $\left(x_{k},z_{k}\right)$ and $\left(x_{a},z_{a}\right)$, respectively. The specific calculation is as denoted in Equation (1):

(1) $$\begin{array}{cc} q_{1} & =atan(\frac{x_{k}-x_{h}}{z_{h}-z_{k}}),\\ q_{2} & =q_{1}-atan\left(\frac{x_{a}-x_{k}}{z_{k}-z_{a}}\right). \end{array}$$

**Figure 4:**
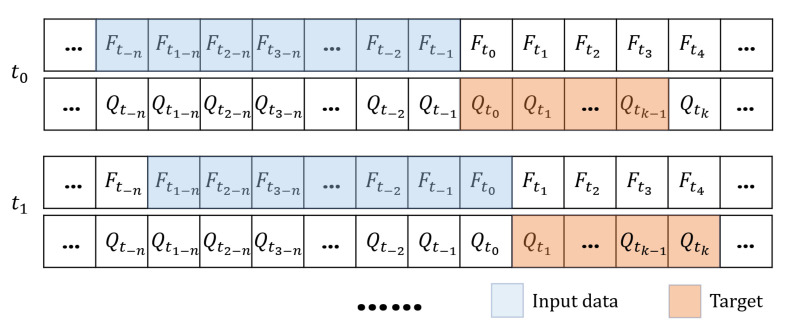
Moving-window-based data division.

Assume that the current moment is $t_{0}$ andthe plantar pressure and joint angles are denoted as $F_{t_{0}}$ and $Q_{t_{0}}$, respectively (Equation (2)), where $f1_{x}^{t_{0}},f1_{y}^{t_{0}},f1_{z}^{t_{0}}$ represent the plantar pressure of left foot on the *x*, *y* and *z* axes, and $f2_{x}^{t_{0}},f2_{y}^{t_{0}},f2_{z}^{t_{0}}$ represent the right. $q_{1}^{t_{0}},q_{2}^{t_{0}},q_{3}^{t_{0}},q_{4}^{t_{0}}$ denote the joint angles of the left hip joint, left knee joint, right hip joint and right knee joint. In each prediction, the plantar pressure during the previous *n* sampling time was used to predict the joint angles during the next *k* sampling time. Namely, $\left[Q_{t_{0}},\cdots ,Q_{t_{k}}\right]$ was predicted based on $\left[F_{t_{-n}},F_{t_{-n+1}},\cdots ,F_{t_{-2}},F_{t_{-1}}\right]$. For the training of the model, $\left[Q_{t_{0}},\cdots ,Q_{t_{k}}\right]$ is the target and $\left[F_{t_{-n}},F_{t_{-n+1}},\cdots ,F_{t_{-2}},F_{t_{-1}}\right]$ are the input data. Therefore, we further divide data via the moving window approach. The details are presented in Figure 4.

(2) $$\begin{array}{cc} F_{t_{0}} & ={\left[f1_{x}^{t_{0}},f1_{y}^{t_{0}},f1_{z}^{t_{0}},f2_{x}^{t_{0}},f2_{y}^{t_{0}},f2_{z}^{t_{0}}\right]}^{T}\\ Q_{t_{0}} & ={\left[q_{1}^{t_{0}},q_{2}^{t_{0}},q_{3}^{t_{0}},q_{4}^{t_{0}}\right]}^{T}. \end{array}$$

### 3.2. Variational Mode Decomposition

Decomposing data according to frequency domain characters is capable of distinctly distinguishing the trend, periodicity and noise that are mixed in the time domain. In the process of obtaining the decomposed components, VMD determines the frequency center and bandwidth of each component by iteratively searching for the optimal solution of the variational model, which enables us to segment the frequency domain of the signal adaptively and separates each component effectively.

VMD decomposes the input signal *S* into *K* intrinsic node functions $U_{k}\left(t\right),k\in \left[1,K\right]$. The constrained variational problem is described in Equation (3):

(3) $$\begin{array}{c} \min_{\left\{U_{k}\right\},\left\{\omega_{k}\right\}}\sum_{k}{∥\partial_{t}\left[\left(\delta \left(t\right)+\frac{j}{\pi t}\right)*U_{k}\left(t\right)\right]e^{-j\omega_{k}t}∥}_{2}^{2}\\ \;s.t\;\sum_{k}U_{k}=S. \end{array}$$

The constraint means that the sum of all modes is equal to the original signal. The symbol ∂(t) denotes the partial derivative operation on time. δ(t) refers to the Dirac distribution. $U_{k}$ and $\omega_{k}$ denote the set of all modes and the set of central frequencies corresponding to these modes. To solve the constrained variational problem, a quadratic penalty term and Lagrange multipliers are introduced to transform the problem into an unconstrained problem in VMD (Equation (4)).

(4) $$\begin{array}{c} L\left(\left\{U_{k}\right\},\left\{\omega_{k}\right\},\lambda \right)=\alpha \sum_{k}{∥\partial_{t}\left[\left(\delta \left(t\right)+\frac{j}{\pi t}\right)*U_{k}\left(t\right)\right]e^{-j\omega_{k}t}∥}_{2}^{2}+∥S\left(t\right)-\sum_{k=1}^{K}U_{k}\left(t\right)∥\\ +\langle \lambda \left(t\right),S\left(t\right)-\sum_{k=1}^{K}U_{k}\left(t\right)\rangle , \end{array}$$

where α is the penalty parameter and λ is the Lagrangian multiplier. $U_{k}\left(t\right)$, $\omega_{k}$ and λ are updated by the Altering Direction Multiplier Method (Equation (5)).

(5) $$\begin{array}{c} {\hat{U}}_{k}^{n+1}\left(\omega \right)=\frac{\hat{S}\left(\omega \right)-\sum_{i\neq k}{\hat{U}}_{i}\left(\omega \right)+\frac{\hat{\lambda }\left(\omega \right)}{2}}{1+2\alpha {\left(\omega -\omega_{k}\right)}^{2}},\\ \omega_{k}^{n+1}=\frac{\int_{0}^{\infty }\omega {\left|{\hat{U}}_{k}\left(\omega \right)\right|}^{2}d\omega }{\int_{0}^{\infty }{\left|{\hat{U}}_{k}\left(\omega \right)\right|}^{2}d\omega },\\ {\hat{\lambda }}^{n+1}\left(\omega \right)={\hat{\lambda }}^{n}\left(\omega \right)+\tau \left(\hat{S}\left(\omega \right)-\sum_{k}{\hat{U}}_{k}^{n+1}\left(\omega \right)\right), \end{array}$$

where ${\hat{U}}_{k}^{n+1}\left(\omega \right)$, $\hat{S}\left(\omega \right)$, ${\hat{U}}_{i}\left(\omega \right)$, ${\hat{\lambda }}^{n+1}\left(\omega \right)$, $\hat{S}\left(\omega \right)$ are the Fourier transforms of the corresponding components. *n* represents the number of iterations in the solution process. Based on the above solution formula, the decomposed modes are obtained.

### 3.3. Basic Transformer Architecture

The transformer adopts three foundational components: positional encoding, a multi-head attention mechanism and a feed-forward network. The three modules endow the transformer with a strong feature processing ability for sequence tasks and provide the backbone of our model.

#### 3.3.1. Multi-Head Attention Mechanism

The attention mechanism is the core component of a transformer. Self-attention captures information about the whole segment of data and learns correlations between different parts. The self-attention mechanism used in the transformer is Scaled Dot-Product Attention. The computation equation is set as follows:

(6) $$\mathrm{Attention}\left(Q,K,V\right)=\mathrm{softmax}\left(\frac{QK^{T}}{\sqrt{d_{k}}}\right)V$$

where *Q*, *K* and *V* are obtained by a linear operation based on the same input.

In order to learn deeper and more diverse features, multi-head self-attention is applied in the transformer.

(7) $$\begin{array}{cc} \mathrm{MultiHead}(Q,K,V) & =\mathrm{Concat}\left({\mathrm{head}}_{1},\ldots ,{\mathrm{head}}_{h}\right)W^{O},\\ {\mathrm{head}}_{i} & =\mathrm{Attention}\left(QW_{i}^{Q},KW_{i}^{K},VW_{i}^{V}\right), \end{array}$$

where h is the number of attention heads and i∈(1,h). ${W_{i}}^{Q}$, ${W_{i}}^{K}$, ${W_{i}}^{V}$ and $W^{O}$ can be regarded as the weight matrices of the linear operation.

#### 3.3.2. Feed-Forward Network

The feed-forward network comprises two fully connected layers followed by an activation layer. It is defined in Equation (8):

(8) $$\mathrm{FFN}\left(x\right)=\max\left(0,xW_{1}+b_{1}\right)W_{2}+b_{2}$$

where $W_{1}$, $W_{2}$ and $b_{1}$, $b_{2}$ denote the weight matrices and bias of linear operation, respectively. max(0,·) refers to the ReLU function. A feed-forward module further integrates the features and enhances the nonlinearity of the network.

#### 3.3.3. Positional Encoding

To handle the problem that the chronological information will be weakened by parallel input operations, the transformer proposes a position encoding method using the properties of sine and cosine functions to retain relative and absolute position information about the input data (Equation (9)).

(9) $$\begin{array}{cc} PE_{\left(pos,2i\right)} & =\sin\left(pos/10000^{2i/d_{\mathrm{model}}}\right),\\ PE_{\left(\mathrm{pos}\;,2i+1\right)} & =\cos\left(pos/10000^{2i/d_{\mathrm{model}}}\right). \end{array}$$

For plantar force data, pos indicates the moment that data are located in temporal dimension, and *i* means the position of features encoded at each moment. $d_{\mathrm{model}}$ denotes the size of the force-space dimension. This method guarantees the uniqueness of each location code value, the consistency of the distance between adjacent location code values and the adaptability to the length of the data.

### 3.4. Convolutional Neural Networks

A CNN is a kind of feed-forward neural network with a deep structure. The convolution operation is the core part of a CNN, which denotes performing inner product operations on local input data and filters. After calculating the local input data within a data window, the data window shifts and slides along the given direction and stride until all data are calculated. The direction and dimensionality of the convolutional operation affect the data features learned by the convolutional layers.

## 4. The Gait Prediction Model

**Figure 5:**
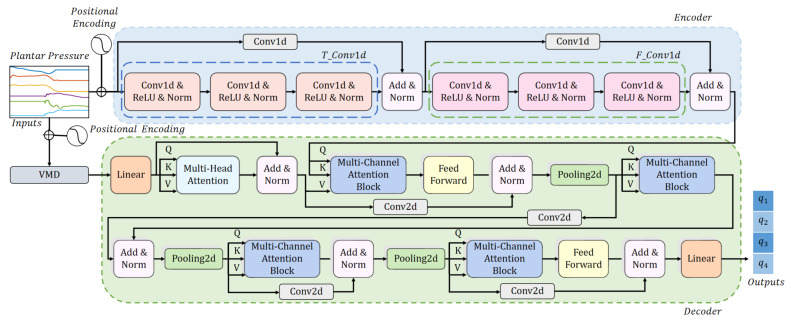
Architecture of the gait prediction model.

Based on the idea that learning characters form different dimensions of data and exploring the relationship in different dimensions are two effective ways of extracting valid information, and combining the advantages of the methods introduced in Section 3, we propose the gait prediction model (TFSformer) to integrate the information in plantar pressure data and predict joint angles. As Figure 5 illustrates, the model adjusts the transformer architecture while retaining the “sequence-to-sequence” structure. For the encoder part, the input data are plantar pressure data with positional encoding, and 1D convolution is utilized to construct this part. For the decoder part, the data decomposed by VMD are applied as the input. To merge the information in different dimensions, we propose multi-channel attention and a deep-multi-channel attention structure based on a multi-head self-attention mechanism. The details of the model are described as follows.

### 4.1. One-Dimensional Convolution-Based Encoder

The force data involve two dimensions: the temporal and force-space dimensions. To uncover both the temporal features and the relationships between forces in different axes, 1D convolution operations are applied in different directions concerning both dimensions of the data, which allows the network to learn features from different perspectives. By utilizing this approach, we are able to extract the most relevant and informative features from the force data, which are then used for further analysis.

**Figure 6:**
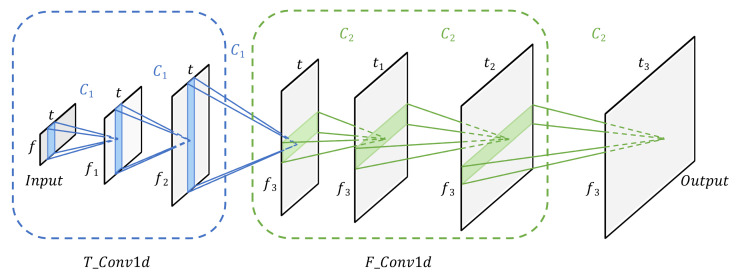
Convolutional diagram of the encoder module. $C_{1}$ represents the one-dimensional convolutional operation performed along the temporal dimension and $C_{2}$ denotes the one-dimensional convolutional operation performed along the force-space dimension. $\left[t,t_{1},t_{2},t_{3}\right]$ and $\left[f,f_{1},f_{2},f_{3}\right]$ represent the lengths of the temporal and force-space dimensions, respectively. In our research, *t*, $t_{1}$, $t_{2}$ and $t_{3}$ are set as 32, 64, 128 and 256, respectively, and $f,\;f_{1},\;f_{2}$ and $f_{3}$ are designed as 6, 36, 108 and 256 in turn.

As shown in Figure 6, the inputs of the encoder part are the raw data with positional encoding. Convolution operations are performed along the temporal dimension first and then along the force-space dimension, expanding the data dimensions while learning features from different dimensions. Specifically, the kernel sizes of the convolution layers in T_Conv1D and F_Conv1D are all set to 3, and the stride and padding are set as 1. Furthermore, as described in Figure 5, residual connections around T_Conv1d and F_Conv1d are employed. Convolutional layers with both the kernel size and stride set to 1 are utilized to adjust the dimension size. In addition, to tune the data distribution and accelerate the convergence speed, we employ a batch normalization layer after the active layers.

### 4.2. Multi-Channel Attention-Based Decoder

**Figure 7:**
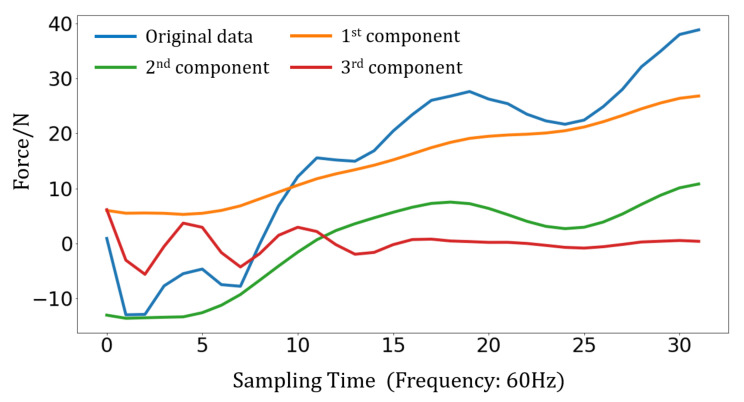
Decomposed modes.

The original data are decomposed by VMD before being input into the decoder. For plantar pressure data decomposition, *k* in Section 3.2 is set as 3. As Figure 7 shows, the high frequency decomposed mode (third component) displays the rapidly changing part of the original data. The other decomposed modes (first and second component) review the overall trends of the original data.

In contrast to the structure of the encoder, the decoder is designed based on an attention mechanism. The first linear layer in the decoder is utilized to expand the width and length of the input data. Then, multi-head self-attention, mentioned in Section 3.3.1, is used to learn the relationships between different parts of the data in the temporal dimension. To further extract features from the two dimensions simultaneously, multi-channel attention and a deep multi-channel attention structure are designed. In addition, the feed-forward layers introduced in Section 3.3.2 are employed to enhance the nonlinearity of the model and tune the weight of data.

#### 4.2.1. Multi-Channel Attention

In multi-head attention, the matrices *Q*, *K* and *V* are obtained by the linear operation performed on the last dimension of the input. The linear layers are capable of remapping and weighting local features of the data, while being relatively weaker at extracting features compared to the convolutional layers. In contrast to 1D convolution, 2D convolution learns information in two dimensions at the same time, which matches the purpose of exploring the relationship between the temporal and force-space dimensions. However, the dimensionality of time series limits the application of 2D convolution.

In view of these two problems, we propose multi-channel attention. As shown in Equation (10), the data are extended by one dimension first. The extended dimension is defined as the channel. Then, 2D convolution is utilized to learn deep, varied information from the temporal and force-space dimensions of the data. The size of the channel dimension is incrementally increased by the convolution operation. Each sublayer in the channel dimension is considered as a single attention head. Multi-channel attention not only enhances the nonlinearity of the model but facilitates feature fusion and extraction in both dimensions.

(10) $$\begin{array}{c} Q_{c}=Conv2d\left(Q.unsqueeze\left(1\right)\right)\\ K_{c}=Conv2d\left(K.unsqueeze\left(1\right)\right)\\ V_{c}=Conv2d\left(V.unsqueeze\left(1\right)\right)\\ MultiChannel\left(Q_{c},K_{c},V_{c}\right)=\mathrm{softmax}\left(\frac{Q_{c}K_{c}^{T}}{\sqrt{d_{k}}}\right)V_{c} \end{array}$$

#### 4.2.2. Deep Multi-Channel Attention Structure

**Figure 8:**
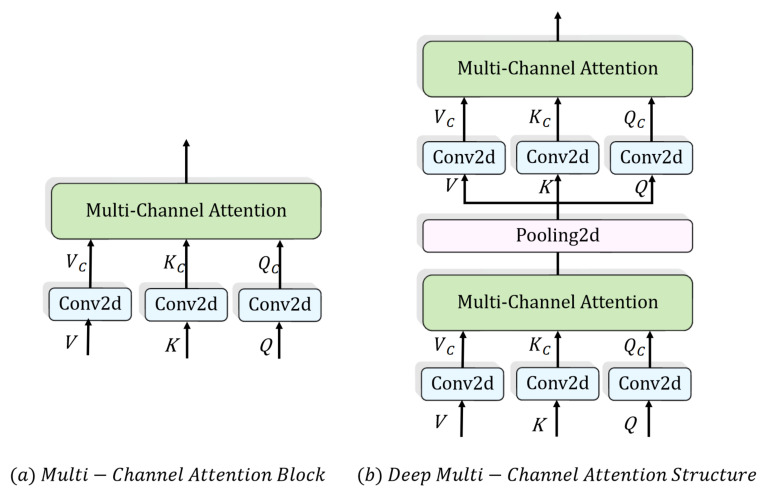
Multi-channel attention.

Inspired by the classical architecture of a CNN, we applied deep multi-channel attention, which increases the channel number while distilling the information in the temporal and variable dimensions. As shown in Figure 8b, a pooling layer is added between two multi-channel attention layers. Pooling layers integrate the features in a small adjacent area, preventing useless parameters from increasing the time complexity on the one hand, and enhancing the integration of features on the other [38].

The specific parameter settings for the decoder module are as follows: (1) the linear layers map the decomposed data dimension to (32,256,256), which is the same as the output of the encoder. (2) The four multi-channel attention blocks deepen the channels in turn to 8, 16, 32 and 64 through 2D convolutional layers. The kernel sizes of these layers are all set to (3,3), the stride is set to (1,1) and padding is denoted as 1. (3) The pooling operation we employed is Maxpooling. The kernel size of the pooling layer is denoted as (2,2), and the stride is set to 2. (4) The output dimensionality of the feed-forward network is the same as the input’s and the dimensionalities of the inner layer are 1024 and 256, respectively.

## 5. Experiment and Results

### 5.1. Experimental Setup

In this section, we present the experimental setup which is fundamental to the experimental design and methodology, including the experimental strategies, dataset partition and hyperparameter settings.

#### 5.1.1. Experimental Strategies

In order to verify the performance of TFSformer, we conducted two experiments: single-step prediction and multi-step prediction. To highlight the superiority of the proposed TFSformer, we made comparisons with three representative neural networks: a CNN, a transformer, and a CNN transformer. The architecture of the comparison models is detailed as follows:

- CNN model: The CNN model is constructed by removing the decoder modules from the proposed TFSformer. Furthermore, the output of the CNN is structured by two linear layers.
- Transformer model: The transformer network for the comparison experiment is derived from the initial transformer architecture developed for a natural language processing (NLP) task in [22]. In contrast to the NLP task, the dimensions of the inputs in our research are fixed. Furthermore, the padding is unnecessary in the experiments. Thus, the primary transformer in [22] is adjusted by excluding the padding mask mechanism. Furthermore, all the information in the inputs is visible at each moment rather than partly invisible as it is in NLP tasks. Thus, the attention-mask mechanism is not employed as well.
- CNN transformer model: The network is constructed on the basis of the transformer network above by replacing the encoder module with a convolutional operation, which is the same as the encoder structure in our proposal. This is to demonstrate the efficiency of the decoder mechanism in TFSformer.

#### 5.1.2. Dataset Partition

Experiments were conducted based on the dataset introduced in Section 3.1. Three volunteers’ data were randomly selected from the dataset as the test set. The data of the remaining 32 people were divided into the training set and the validation set in the ratio of 8:2, respectively.

For the single-step prediction experiment, the parameters *n* and *k* mentioned in Figure 4 were set to 32 and 1, respectively. For the multi-step prediction experiment, we specified two subtasks, namely n=32, k=3 and n=32, k=6. The data capacities for the training, validation and test sets are displayed in Table 1.

#### 5.1.3. Hyperparameter Settings

To ensure the validity of the comparison experiment, we set the same hyperparameters in all experiments. The batch size was set as 32. The initial learning rate was set to 0.0001 and the optimizer we used was AdamW. The loss we used in the training was the mean absolute error (MAE). In addition, both the mean squared error (MSE) and the MAE were set as evaluation indicators (Equation (11)).

(11) $$\begin{array}{c} MSE=\frac{1}{4n}\sum_{i=1}^{n}{\left(Q_{i}-{\hat{Q}}_{i}\right)}^{2},\\ MAE=\frac{1}{4n}\sum_{i=1}^{n}\left|Q_{i}-{\hat{Q}}_{i}\right|. \end{array}$$

### 5.2. Experimental Results

Table 2 displays the experimental results of all models. The best results of each experiment are highlighted in bold. Based on an analysis of the data, the following observations are derived: (1) In the single-step prediction task, the mean MSE of TFSformer decreases by 10.83%, 15.04% and 4.74% and the mean MAE decreases by 20.40%, 29.09% and 10.43% compared with the CNN, transformer, and CNN transformer, respectively. (2) In the multi-step prediction task with k=3, TFSformer achieves the lowest MAE. On the other hand, the MSE of TFSformer outperforms that of the CNN and CNN transformer, while it is 5.06% higher than the transformer. (3) In the case of k=6, TFSformer yields higher than 5.47% MAE and 4.00% MSE reductions compared with the other models.

Further conclusions are drawn as follows:

- The transformer model exhibited an inferior performance in single-step prediction, while the CNN model demonstrated a weaker prediction ability compared to the other three models in multi-step prediction tasks. These results suggest that each individual approach, either a convolutional operation or an attention mechanism, has limitations. Furthermore, combining the strengths of both approaches contributes to obvious improvements in the network’s ability to capture temporal features.
- The phenomenon that TFSformer outperforms the CNN transformer in all tasks fully demonstrates the superiority of the multi-channel attention-based decoder mechanism in our proposal.

In conclusion, TFSformer has a superior predictive ability, particularly for hip motion prediction, and though the MES and MAS are slightly larger in knee joint prediction, the errors are within acceptable limits. Additionally, the prediction errors of knee joints are slightly higher than hip joints due to the significant personal difference in knee joint movements during walking.

**Figure 9:**
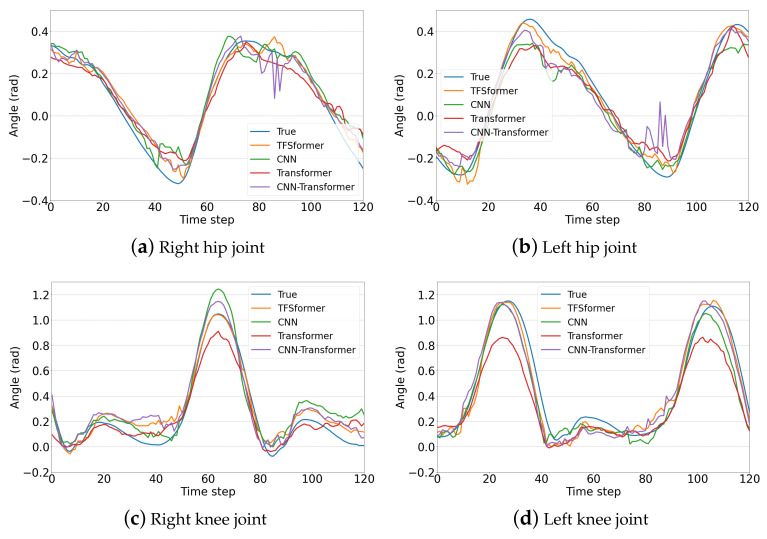
Gait prediction results on the data of subject 1.

**Figure 10:**
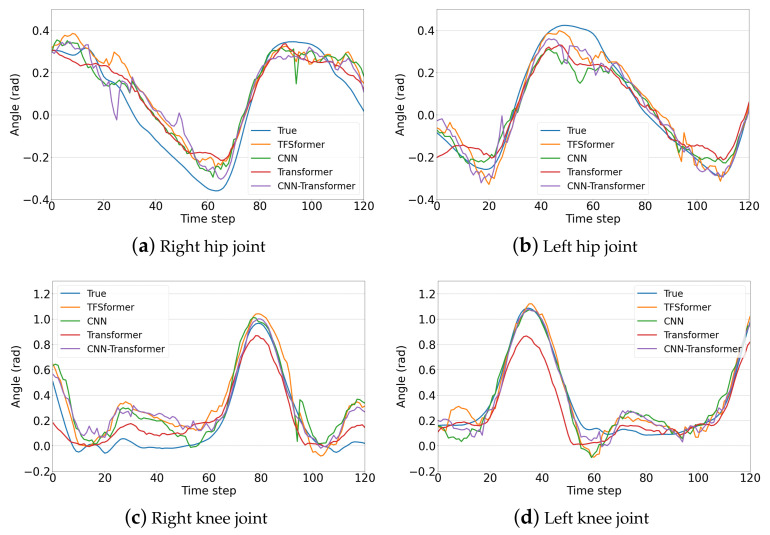
Gait prediction results on the data of subject 2.

**Figure 11:**
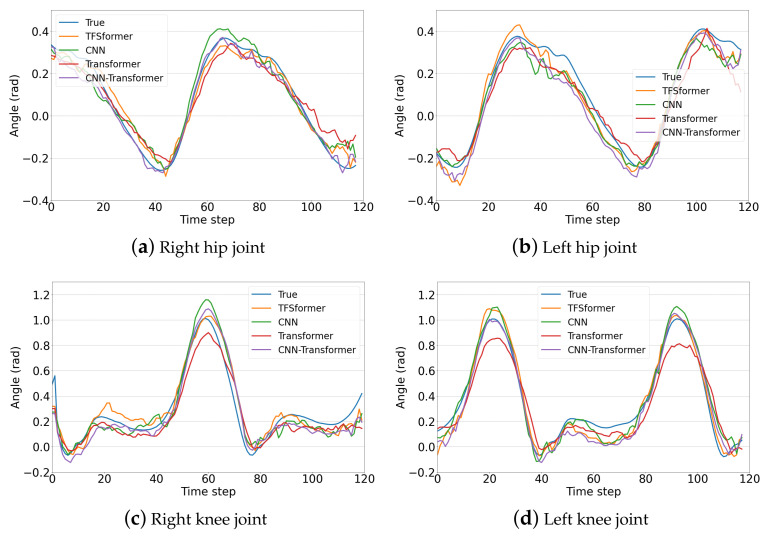
Gait prediction results on the data of subject 3.

Taking into account differences in gait due to physical metrics and individual habits, we took data from about one gait period from the results obtained based on the data of three subjects in the single-step prediction task to further compare and analyze the performance of different models. The basic information of the subjects is shown in Table 3, and the prediction results of subject 1, subject 2 and subject 3 are displayed in Figure 9, Figure 10 and Figure 11. Subgraphs (a)–(d) demonstrate the results of the right hip joint, left hip joint, right knee joint and left knee joint, respectively. From the figures, it is noticeable that:

- TFSformer (the orange line) is capable of capturing the change in joint angles around poles well, while the errors of the CNN (the green line) and the transformer (the red line) are slightly larger.
- In Figure 9a,b and Figure 10, apparent oscillations are observed in the prediction values of the CNN transformer (the purple line).
- During the interval within the gentle angle change, the prediction value of TFSformer is relatively smooth and in line with the true changing trends.

According to the analysis above, the proposed model TFSformer exhibits the best comprehensive performance. Except for the transformer model in the multi-step prediction task when k=3, TFSformer achieves the highest average accuracy in all prediction tasks. Even for multi-step prediction tasks when k=3, TFSformer obtains the lowest MAE. More importantly, TFSfomer demonstrates a strong robustness and stability, predicting the gait trajectory of different people with small errors regardless of individual gait diversity.

## 6. Conclusions

In this paper, we propose a transformer-based model, TFSformer, to predict bilateral hip joint and knee joint angles through the plantar pressure. Specifically, we design a 1D convolution-based encoder to learn features in different dimensions and use multi-channel attention to extract characteristics from temporal and force-space dimensions simultaneously. Furthermore, a deep multi-channel attention structure is employed to reduce the memory and computing consumption. In addition, VMD is utilized to discompose the data while retaining the original information to distinguish trends and variations in the data. Finally, to verify the performance of the proposed method, we built a gait capture platform and constructed a gait dataset with data from 35 volunteers. Based on this gait dataset, we conducted comparative experiments. Although FTSformer does not achieve the optimal performance with overwhelming superiority in all tasks, the results show that FTSformer exhibits the strongest comprehensive performance, not only predicting joint angles accurately in both multi-step prediction and single-step prediction tasks, but featuring a high robustness regardless of gait diversity. In practical applications, the proposal is capable of compensating for sensor delays and providing references for controllers, thus improving collaboration between humans and robots. In the future, we plan to further optimize the model and work on integrating the gait prediction model with controller design to further confirm its validity.

## Figures and Tables

**Table 1:** Experimental data capacity.

	Single-Step Prediction (*k* = 1)	Multi-Step Prediction (*k* = 3)	Multi-Step Prediction (*k* = 6)
Training set	40,864	40,234	39,289
Validation set	8392	8362	8167
Test set	6150	6058	5920

**Table 2:** Experimental results.

Models		TFSformer		CNN		Transformer		CNN-Transformer	
**Metric**		**MAE**	**MSE**	**MAE**	**MSE**	**MAE**	**MSE**	**MAE**	**MSE**
Task1	$H_{l}$	**0.0525**	**0.0035**	0.0645	0.0070	0.0667	0.0065	0.0590	0.0063
	$H_{r}$	**0.0479**	**0.0043**	0.0614	0.0066	0.0664	0.0070	0.0579	0.0062
	$K_{l}$	**0.0725**	**0.0085**	0.0818	0.0112	0.1053	0.0196	0.0743	0.0089
	$K_{r}$	0.1004	0.0148	0.0989	0.0146	**0.0831**	**0.0108**	0.0956	0.0135
	*M*	**0.0683**	**0.0078**	0.0766	0.0098	0.0804	0.0110	0.0717	0.0087
Task2	$H_{l}$	**0.0487**	**0.0038**	0.0602	0.0060	0.0542	0.0046	0.0581	0.0061
	$H_{r}$	0.0523	0.0043	0.0598	0.0061	**0.0506**	**0.0040**	0.0617	0.0071
	$K_{l}$	0.0805	0.0112	0.0833	0.0120	0.0873	0.0127	**0.0777**	**0.0101**
	$K_{r}$	0.0925	0.0137	0.0989	0.0150	**0.0829**	**0.0103**	0.1005	0.0149
	*M*	**0.0685**	0.0083	0.0756	0.0098	0.0689	**0.0079**	0.0745	0.0095
Task3	$H_{l}$	**0.0449**	**0.0048**	0.0549	0.0050	0.0520	0.0043	0.0522	0.0050
	$H_{r}$	0.0559	**0.0032**	0.0643	0.0067	**0.0543**	0.0044	0.0574	0.0060
	$K_{l}$	0.0841	0.0113	**0.0721**	**0.0088**	0.0801	0.0108	0.0790	0.0109
	$K_{r}$	**0.0858**	**0.0106**	0.1075	0.0175	0.0889	0.0119	0.0979	0.1412
	*M*	**0.0677**	**0.0075**	0.0747	0.0095	0.0688	0.0078	0.0716	0.0090

Task1, Task2 and Task3 represent the single-step prediction task (k=1), multi-step prediction task (k=3) and multi-step prediction task (k=6) in turn. $H_{l}$, $H_{r}$, $K_{l}$, $K_{r}$ and *M* represent the left hip joint, right hip joint, left knee joint, right knee joint and average value, respectively. The values in bold denote the best results of each experiment.

**Table 3:** Basic information of subjects.

	Height (cm)	Weight (kg)	Thigh Length (cm)	Shank Length (cm)	Gender	Age
Subject 1	178.0	68.3	40.2	40.3	male	27
Subject 2	164.1	58.2	36.5	36.2	female	24
Subject 3	176.3	74.5	38.9	40.1	male	26

## Data Availability

The data are not publicly unavailable due to ethical and privacy issues.
